# Insufficient Antigen Presentation Due to Viral Immune Evasion Explains Lethal Cytomegalovirus Organ Disease After Allogeneic Hematopoietic Cell Transplantation

**DOI:** 10.3389/fcimb.2020.00157

**Published:** 2020-04-15

**Authors:** Rafaela Holtappels, Sina I. Schader, Oliver Oettel, Jürgen Podlech, Christof K. Seckert, Matthias J. Reddehase, Niels A. W. Lemmermann

**Affiliations:** Institute for Virology and Research Center for Immunotherapy (FZI) at the University Medical Center of the Johannes Gutenberg-University of Mainz, Mainz, Germany

**Keywords:** avidity, bone marrow transplantation, CD8 T cells, Graft-vs.-host (GvH) reaction, hematopoietic reconstitution, host-vs.-graft (HvG) reaction, murine cytomegalovirus, nodular inflammatory focus (NIF)

## Abstract

Reactivation of latent cytomegalovirus (CMV) poses a clinical problem in transiently immunocompromised recipients of hematopoietic cell (HC) transplantation (HCT) by viral histopathology that results in multiple organ manifestations. Compared to autologous HCT and to syngeneic HCT performed with identical twins as HC donor and recipient, lethal outcome of CMV infection is more frequent in allogeneic HCT with MHC/HLA or minor histocompatibility loci mismatch between donor and recipient. It is an open question if a graft-vs.-host (GvH) reaction exacerbates CMV disease, or if CMV exacerbates GvH disease (GvHD), or if interference is mutual. Here we have used a mouse model of experimental HCT and murine CMV (mCMV) infection with an MHC class-I mismatch by gene deletion, so that either HCT donor or recipient lack a single MHC class-I molecule, specifically H-2 L^d^. This particular immunogenetic disparity has the additional advantage that it allows to experimentally separate GvH reaction of donor-derived T cells against recipient's tissues from host-vs.-graft (HvG) reaction of residual recipient-derived T cells against the transplanted HC and their progeny. While in HvG-HCT with L^d^-plus donors and L^d^-minus recipients almost all infected recipients were found to control the infection and survived, almost all infected recipients died of uncontrolled virus replication and consequent multiple-organ viral histopathology in case of GvH-HCT with L^d^-minus donors and L^d^-plus recipients. Unexpectedly, although anti-L^d^-reactive CD8^+^ T cells were detected, mortality was not found to be associated with GvHD histopathology. By comparing HvG-HCT and GvH-HCT, investigation into the mechanism revealed an inefficient reconstitution of antiviral high-avidity CD8^+^ T cells, associated with lack of formation of protective nodular inflammatory foci (NIF) in host tissue, selectively in GvH-HCT. Most notably, mice infected with an immune evasion gene deletion mutant of mCMV survived under otherwise identical GvH-HCT conditions. Survival was associated with enhanced antigen presentation and formation of protective NIF by antiviral CD8^+^ T cells that control the infection and prevent viral histopathology. This is an impressive example of lethal viral disease in HCT recipients based on a failure of the immune control of CMV infection due to viral immune evasion in concert with an MHC class-I mismatch.

## Introduction

Hematopoietic cell transplantation (HCT) is the only curative therapeutic option in the treatment of hematopoietic malignancies that are resistant to standard therapies. The aim of HCT is to replace the patient's hematopoietic system with hematopoietic cells (HC) derived from a healthy donor. This is achieved by hematoablative treatment followed by HCT. This treatment is inherently associated with a “window of risk” based on transient immunodeficiency until the transplanted hematopoietic stem cells and progenitor cells have reconstituted the immune system of the recipient (Maury et al., 2001).

Major complications in the therapy by HCT include “minimal residual disease/leukemia” (MRD/L) that can lead to leukemia relapse, graft-vs.-host disease (GvHD) in cases of immunogenetic MHC/HLA or minor histocompatibility antigen mismatch (Singh and McGuirk, 2016) and opportunistic infections (Sahin et al., 2016; Schuster et al., 2017). Infectious complications profit from the transient immunodeficiency inherent to HCT as well as from an immunosuppressive therapy of GvHD. Current research and clinical trials aim at precluding GvHD, while retaining beneficial graft-vs.-leukemia (GvL) and graft-vs.-infection (GvI) functions of the HC transplant or of a donor lymphocyte infusion (DLI) (Chopra et al., 2016; Singh and McGuirk, 2016). Based on promising clinical trials, adoptive transfer of virus-specific immune cells is an advanced approach to control the infection and prevent viral histopathology before hematopoietic reconstitution by HCT takes over (Moss and Rickinson, 2005).

Among viral complications in HCT patients, infection with human cytomegalovirus (hCMV) is the most frequent and most feared. It can lead to lethal organ disease, in particular to interstitial pneumonia, unless infection is treated by pre-emptive antiviral chemotherapy as soon as diagnosed in the routine follow-up monitoring (Hebart and Einsele, 2004; Seo and Boeckh, 2013; Stern et al., 2019). As antivirals have adverse off-target effects, including inhibition of hematopoietic reconstitution, adoptive immunotherapy of hCMV infection by immune cell transfer is used in clinical trials as a potentially less burdening alternative (Riddell et al., 1992; Walter et al., 1995; Moss and Rickinson, 2005; Feuchtinger et al., 2010; Neuenhahn et al., 2017). Except in rare and rather accidental cases, hCMV infection in HCT recipients is not caused by transmission of infectious virus from a virus-shedding contact person or by an acutely infected transplant. Instead, it results from reactivation of latent virus present in HC of a latently infected donor or, more frequently, of latent virus harbored already pre-HCT in cells of the recipient (Emery, 1998; Stern et al., 2019; for a review and discussion, see Reddehase and Lemmermann, 2019). Notably, the incidence of hCMV organ disease is more frequent in allogeneic HCT with family donors or unrelated donors, compared to syngeneic HCT with identical twins as donor and recipient (Applebaum et al., 1982; Meyers et al., 1982) or to autologous HCT (Wingard et al., 1988). This suggests a pathogenetic link between hCMV disease and a donor-recipient mismatch in MHC/HLA antigens and/or non-MHC/HLA minor histocompatibility antigens.

As clinical studies exclude experimental approaches and as host-species specificity of CMVs precludes studying hCMV in animal models, except for specific questions in humanized mouse models (Crawford et al., 2015; Thomas et al., 2015; Caposio et al., 2019; Wahl et al., 2019), the mouse model of studying murine cytomegalovirus (mCMV) in its natural host is well-established and has proven its validity in revealing the more general principles of CMV-host interactions. Specifically, CMV pathogenesis/disease, immune control, and cell-based immunotherapy in the mouse model were of predictive value as confirmed later on by clinical investigation (reviewed in Reddehase and Lemmermann, 2018). A focus of previous research in our group has been to establish a mouse model of experimental syngeneic HCT and mCMV infection, using susceptible BALB/c mice (MHC haplotype *H-2*^*d*^) as HC donors and recipients. We had chosen this focus on purpose to understand this basal HCT setting before introducing a further layer of complication by immunogenetic donor-recipient mismatch. In essence, the previous studies have shown that HCT variables such as the degree of hematoablation of the recipients and the numbers of transplanted donor HC reciprocally determine the clinical outcome of infection, ranging from survival to death of the HCT recipients. Efficient and timely reconstitution of antiviral CD8^+^ T cells proved to be the decisive parameter for confining infection to protective nodular inflammatory foci (NIF). These are microanatomical structures in host tissues where antiviral CD8^+^ T cells recognize infected tissue cells to limit viral intra-tissue spread and thus prevent extensive viral histopathology (Holtappels et al., 2013; Reddehase, 2016; Reddehase and Lemmermann, 2018).

Here we have extended this model by introducing a singular MHC class-I disparity, namely expression or absence of the L^d^ molecule in BALB/c mice and the congenic *L*^*d*^ gene deletion mutant BALB/c-H-2^dm2^, respectively. This specific immunogenetic constellation prevents bidirectional GvH and host-vs.-graft (HvG) reactivity against L^d^, thereby separating GvH-HCT (donor BALB/c-H-2^dm2^, recipient BALB/c) from HvG-HCT (donor BALB/c, recipient BALB/c-H-2^dm2^). Remarkably, our data show that infection is controlled in the HvG setting, whereas lethal disease occurs selectively in the GvH setting. The cause of death in GvH-HCT proved not to be an exacerbation of GvHD by factors associated with infection, as one might have presumed. Instead, lethal disease is found to be associated with a failure in the reconstitution and tissue recruitment of high-avidity antiviral CD8^+^ T cells for NIF formation, resulting in extensive viral histopathology caused by an uncontrolled virus spread. Most notably, under otherwise identical conditions of GvH-HCT, improved antigen presentation by deletion of viral immune evasion genes restored control of infection within NIF and prevented lethal CMV disease.

## Materials and Methods

### Mice and Cell Lines

BALB/cJ (*H-2 K*^*d*^*, -D*^*d*^*, -L*^*d*^) and congenic BALB/c-H-2^dm2^ (*H-2 K*^*d*^*, -D*^*d*^; Rubocki et al., 1986) mice were bred and housed under specified-pathogen-free conditions at the Central Laboratory Animal Facility of the Johannes Gutenberg University, Mainz, Germany.

Murine embryonic fibroblasts (MEF) were isolated from BALB/cJ and BALB/c-H-2^dm2^ mice by standard protocol (Podlech et al., 2002) and cultured in MEM/10% FCS. Cells of the murine fibroblast cell line L (ATCC CCL1.3) and of the stably-transfected sub-line L^Ld^ expressing H-2 L^d^ (Ponta et al., 1985) were cultured in DMEM/10% FCS and in DMEM/10% FCS-HAT medium (100 μM Hypoxanthine, 16 μM Thymidine, 0.4 μM Aminopterin), respectively. The TAP-deficient human hybridoma T2 (ATCC CRL-1992) and T2 cells stably-transfected with the *H-2-L*^*d*^ gene (T2^Ld^; Alexander et al., 1989) were cultured in RPMI/10% FCS supplemented with 10 mM HEPES, 2 mM L-glutamine, and 50 mM β-mercaptoethanol. For culturing T2^Ld^ cells, 1 mg/ml G418 was added.

### Viruses and Infection

Intraplantar infection of 8–10 week-old mice was performed at the left hind footpad with 1 × 10^5^ plaque-forming units (PFU) of mCMV (strain Smith, ATCC VR-1399), bacterial artificial chromosome (BAC)-derived mCMV MW97.01 (mCMV-WT.BAC; Wagner et al., 1999), or immunoevasin gene deletion mutant mCMV-Δm04+m06+m152 (mCMV-ΔvRAP; Wagner et al., 2002). Cell culture-derived BAC-free high titer virus stocks were generated by standard protocol (Podlech et al., 2002; Lemmermann et al., 2010).

### Experimental HCT

HCT was performed as described in greater detail previously (Podlech et al., 2002). In essence, HCT recipient mice were subjected to total-body γ-irradiation with a single dose of 6.5 Gy. HCT was performed by infusion of 5 × 10^6^ donor-derived tibial and femoral bone marrow cells into the tail vein of the recipients. Subsequently, the recipients were infected with mCMV.

### Quantification of Hematopoietic Reconstitution and Chimerism

Bone marrow cells were isolated from one tibia, and DNA was extracted with the DNeasy Blood & Tissue kit (Qiagen, Hilden, Germany). Quantification in absolute numbers of the *pthrp* gene and of the Y-chromosomal gene *sry* was performed by SYBR-Green qPCR (Lemmermann et al., 2010), normalized to a log_10_-titrated standard of linearized plasmid pDrive_gB_PTHrP_Tdy (Simon et al., 2005; Lemmermann et al., 2010).

### Immunomagnetical Purification of Liver Tissue-Infiltrating CD8^+^ T Cells

Non-parenchymal liver cells were isolated as described previously (Seckert et al., 2009), and CD8^+^ T cells in the cell suspension were immunomagnetically purified by positive selection with CD8 MicroBeads (Miltenyi Biotec, Bergisch-Gladbach, Germany).

### Assays of CD8^+^ T-Cell Effector Functions

IFNγ-based enzyme-linked immunospot (ELISpot) assays were used to detect sensitization of CD8^+^ T cells by MHC-I-presented synthetic or naturally processed antigenic peptides (Pahl-Seibert et al., 2005; Böhm et al., 2008b, and references therein). In brief, graded numbers of immunomagnetically purified CD8^+^ T cells were sensitized in triplicate assay cultures by incubation with target cells. Frequencies of IFNγ-secreting cells and the corresponding 95% confidence intervals were calculated by intercept-free linear regression analysis. Effector cell stimulation by mCMV-infected cells was performed with MEF that were centrifugally infected with 0.2 PFU per cell (corresponding to a multiplicity of infection of 4; Podlech et al., 2002, and references therein) of the indicated recombinant viruses 90 min before use in the ELISpot assay. IFNγ pretreatment of target cells was performed 48 h prior to infection by incubation in culture medium containing 20 ng of recombinant murine IFNγ (R&D Systems, Minneapolis, MN, USA) per ml. Cytolytic activity of L^d^-reactive T cells was measured in a standard 4-h [^51^Cr]-release assay in triplicate assay cultures with graded numbers of effector cells and 1,000 [^51^Cr]-labeled target cells (L-, L^Ld^-, T2-, and T2^Ld^-cells). Details for the calculation of cytolytic activity were published previously (Holtappels et al., 2006).

### Determination of Functional Avidities

To determine functional avidities of epitope-specific CD8^+^ T cells, target cells were exogenously loaded with synthetic antigenic peptides at graded declining molar concentrations. The spot counts for each peptide concentration quantitate CD8^+^ T cells that respond up to the indicated peptide concentration, which includes cells also responding to lower peptide concentrations. This results in a “cumulative avidity distribution.” For quantitating CD8^+^ T cells that respond precisely to the indicated peptide concentration, which defines their functional avidity, the non-cumulative “Gaussian-like avidity distribution” can be deduced from the cumulative avidity distribution by plotting the differences between spot counts of neighboring peptide concentrations.

### Cytofluorometric Analysis of Vβ Usage

Cytofluorometric analysis of the expression of the TCR β-chain variable region (Vβ) on memory CD8^+^ T cells was performed essentially as described previously (Pahl-Seibert et al., 2005). In brief, CD8^+^ T cells were immunomagnetically purified from the spleen of latently infected mice and unspecific staining was blocked with unconjugated anti-FcγRII/III antibody (anti-CD16/CD32; clone 2.4G2, BD Pharmingen, Heidelberg). Cells were stained with PE-conjugated H-2Ld/YPHFMPTNL (IE1) or APC-conjugated H-2Dd/AGPPRYSRI (m164) MHC class-I multimers (ProImmune, Oxford, UK), and with the mouse Vβ TCR screening panel (BD Pharmingen), which includes FITC-conjugated MAbs directed against mouse Vβ chains (Vβx, where x is 2, 4, 6, 7, 8.1 and 8.2, 8.3, 9, 10^b^, 13, or 14). A gate was set on living cells in the forward-vs.-sideward scatter plot, and Vβx expression was determined either for all CD8^+^ T cells or for multimer-stained, epitope-specific CD8^+^ T cells. Analyses were performed with a FACSort (Becton Dickinson) using CellQuest 3.3 software for data processing.

### Quantitating *in vivo* Infection and Tissue Infiltration of Reconstituted T Cells

At indicated times post-HCT and infection, the load of infectious virus in spleen, lungs, liver, and salivary glands was assessed in the respective organ homogenates as PFU determined by virus plaque assay performed under conditions of centrifugal enhancement of infectivity (see above). Infected cells and T cells in liver tissue sections were detected and quantified by two-color immunohistochemistry (2C-IHC) specific for the intranuclear viral IE1 protein (red staining) and the cell membrane T-cell receptor (TCR) complex molecule CD3ε (black staining) as described in greater detail previously (Podlech et al., 2002; Lemmermann et al., 2010).

### Statistical Analyses

To evaluate statistical significance of differences between two independent sets of log-transformed, log-normally distributed data, the two-sided unpaired *t*-test with Welch's correction of unequal variances was used. Differences were considered as statistically significant for *P*-values of <0.05 (^*^), <0.01 (^**^), and <0.001 (^***^). Kaplan-Meier survival plots were used for documenting survival in independent cohorts. Statistical significance of differences between groups was calculated with log-rank and Gehan-Wilcoxon test. Calculations were performed with GraphPad Prism 6.07 (GraphPad Software, San Diego, CA, USA).

Frequencies of IFNγ-secreting cells responding in the ELISpot assay and the corresponding 95% confidence intervals were calculated by intercept-free linear regression analysis based on spot counts from triplicate assay cultures for each of the graded cell numbers seeded, as described previously (Pahl-Seibert et al., 2005; Böhm et al., 2008b). Calculations were performed with Mathematica, 8.0.4 (Wolfram Research, Champaign, Il, USA).

## Results

### MHC Class-I Mismatch in Concert With CMV Infection Is Lethal Selectively in GvH-HCT

**Figure 1:**
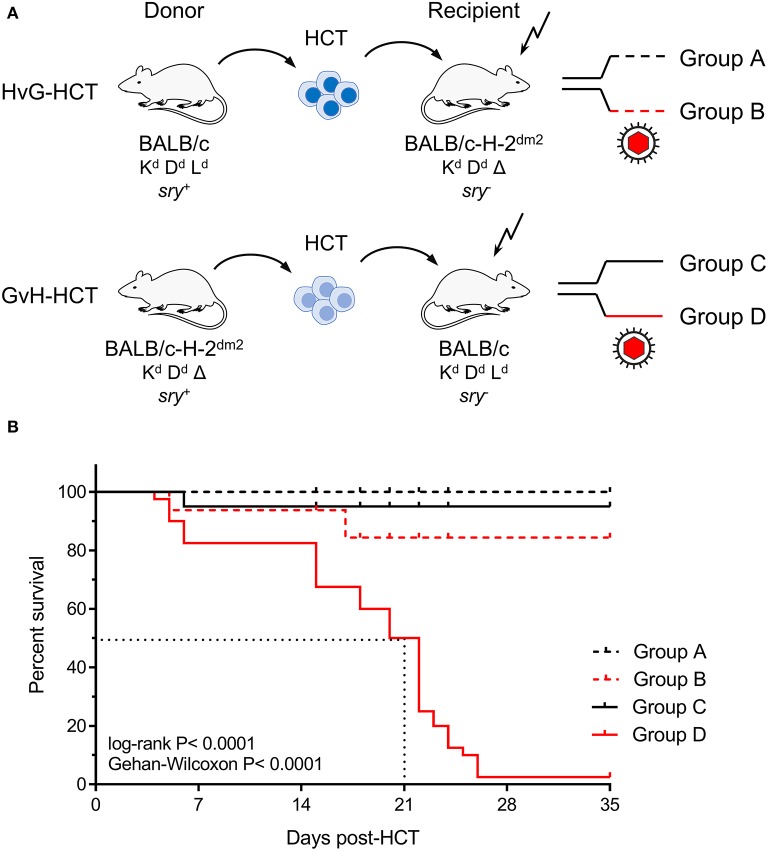
Lethality of infection after GvH-HCT. **(A)** Sketch of the transplantation models used throughout this study and defining groups A–D. The flash symbol indicates hematoablative conditioning of the HCT recipients by total-body γ-irradiation. **(B)** Kaplan-Meyer survival plots. The dotted lines indicate the median survival time of mCMV-infected GvH-HCT recipients. Censured animals are marked.

Experimental HCTs were performed with BALB/c mice (expressing MHC class-I molecules K^d^, D^d^, and L^d^) as HC donors or recipients and the spontaneous loss mutant strain BALB/c-H-2^dm2^ (lacking L^d^, Rubocki et al., 1986) as HC recipients or donors, respectively. The immunogenetic potential for an HvG response of recipient-resident immune cells against L^d^ expressed on HC of the donor defines an HvG-HCT setting, whereas the immunogenetic potential for a GvH response of donor-derived immune cells against L^d^ expressed by cells of the recipient defines a GvH-HCT setting (Figure 1A). On demand, male mice (*sry*^+^) were used as donors to track transplanted HC by detection of the Y-chromosomal gene *sry* (Koopman et al., 1990). Recipients in both HCT settings were either left uninfected or were infected with mCMV.

The outcome revealed a dramatic difference dependent on infection and the direction of transplantation. Essentially all uninfected recipients survived irrespective of the HCT setting. Notably, whereas almost all infected recipients of HvG-HCT also survived, infection combined with GvH-HCT was lethal with a median survival time of only 21 days (Figure 1B). As the technical conditions of HCT and infection were identical in HvG-HCT and GvH-HCT, the selective presence of a singular MHC class-I molecule on cells of the recipient accounts for the fundamentally different clinical outcome.

### Inquiry Into the Cause of Death in GvH-HCT

**Figure 2:**
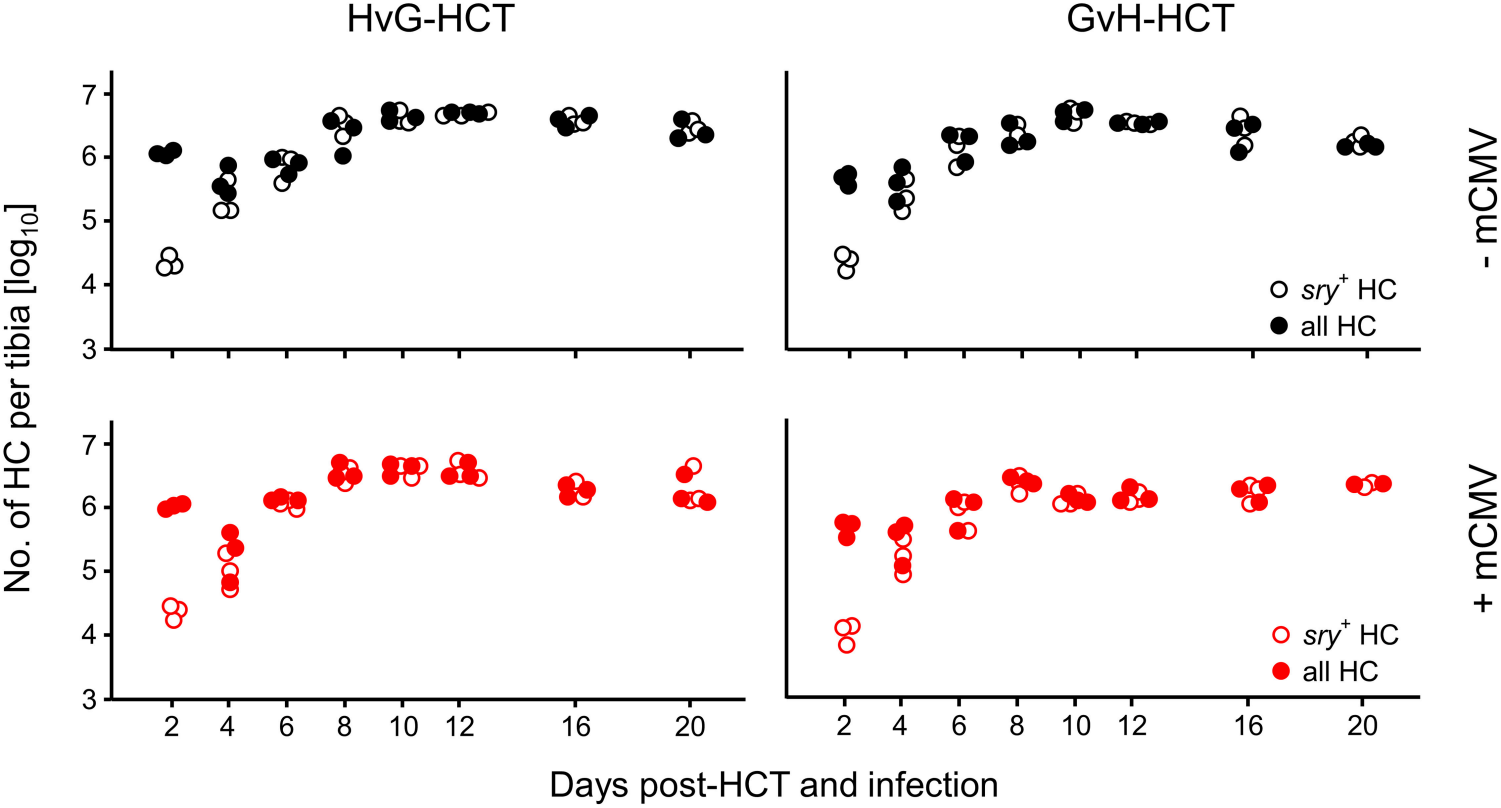
Bone marrow repopulation is not affected by MHC class-I mismatch or mCMV infection. HCT donors were of male sex for tracking the fate of transplanted hematopoietic cells (HC) by qPCR specific for the Y-chromosomal gene *sry*. Donor HC and progeny thereof were quantitated in the tibial bone marrow cell population. Total cell numbers were determined by pthrp-specific qPCR. Symbols indicate mice analysed individually. Note that for comparison of all four HCT groups (recall Figure 1A) data in the lower left panel were reproduced from own previous work on HvG-HCT (Seckert et al., 2011).

We first considered the possibility that transplanted donor hematopoietic stem- and progenitor cells may fail to home to the MHC class-I disparate bone marrow stroma and thus fail to repopulate the emptied bone marrow of the recipients. Such a mechanism would result in an insufficient reconstitution of cells of all hematopoietic lineages. Using male donors and female recipients, quantification of the Y-chromosomal gene *sry* in recipients' bone marrow did not reveal differences in the time course of bone marrow repopulation, regardless of the direction of HCT and regardless of infection. In all experimental groups it took 4 days after hematoablative treatment and HCT to fully replace *sry*^−^ HC of the female recipients with *sry*^+^ HC of the male donors, and plateau levels of repopulation were reached after 8 days (Figure 2).

**Figure 3:**
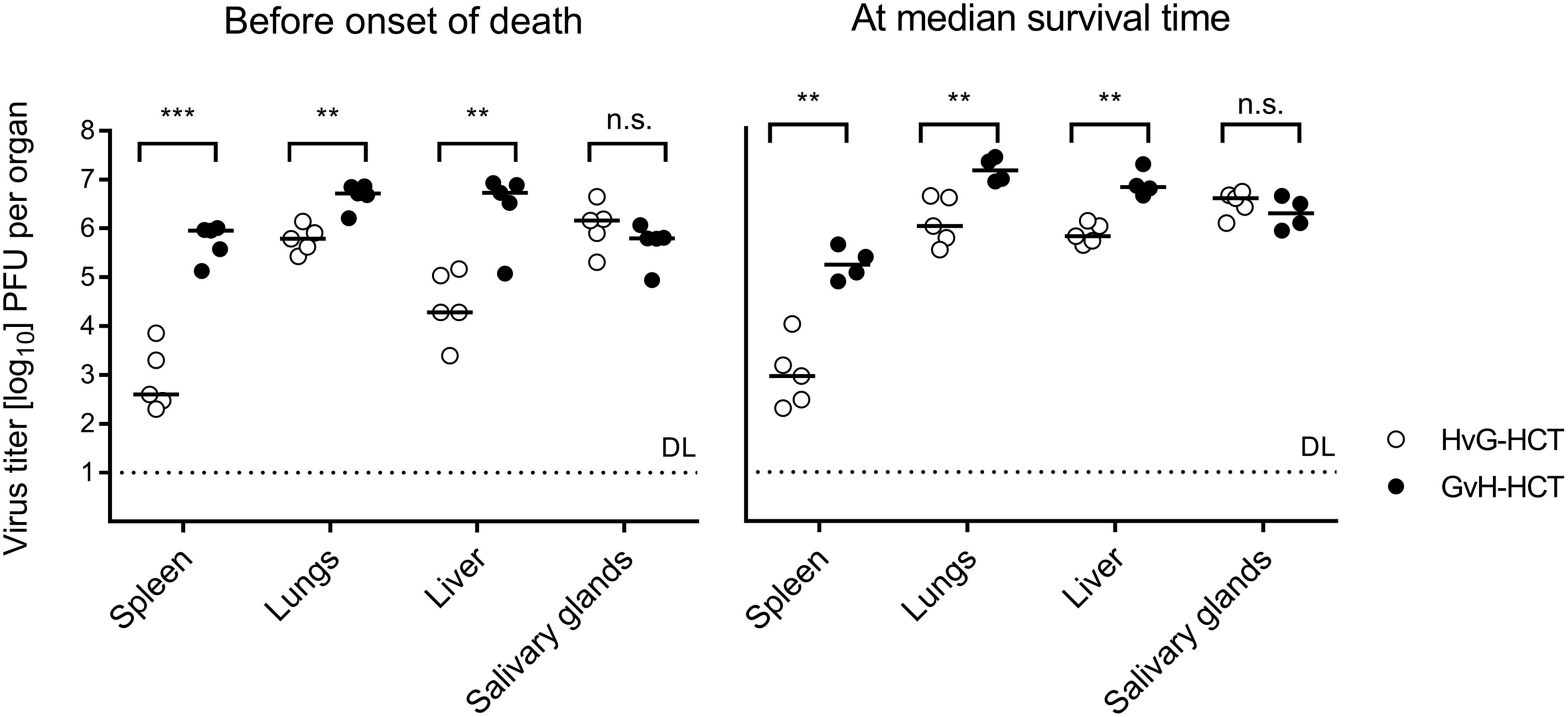
Control of organ infection is diminished in GvH-HCT compared to HvG-HCT. Titers of infectious virus were determined for the indicated organs. (Left panel) Virus replication on day 17, that is shortly before cases of death occur after GvH-HCT. (Right panel) Virus replication on day 21, that is at the median survival time after GvH-HCT (recall Figure 1B). Note that absolute virus titers at the median survival time may be biased by the inherent selection of mice that have survived until day 21. Symbols represent mice analyzed individually. Median values are marked. DL, detection limit of the assay. Differences between two experimental groups were determined by Student's *t*-test based on log-transformed data. Significance levels: *P*-values of <0.01 (**), and <0.001 (***). n.s., not significant.

We next compared virus replication in infected recipients of HvG-HCT and GvH-HCT by quantitating infectious virus in pathogenetically-relevant organs before the onset of death and at the median survival time, as defined in GvH-HCT (Figure 3, recall Figure 1). With salivary glands representing an exception, the viral burden was significantly higher in spleen, lungs, and liver of mice that have undergone GvH-HCT. As CD8^+^ T cells are known to be the principal antiviral effector cells that control infection after syngeneic HCT in organs (Holtappels et al., 1998; Podlech et al., 1998, 2000), this finding was a first hint to suppose a difference between HvG-HCT and GvH-HCT in the reconstitution and/or tissue recruitment of antiviral CD8^+^ T cells. This would also explain why no difference is seen for the salivary glands, the site of persistent virus replication and virus host-to-host transmission, as persistent salivary gland infection is eventually controlled primarily by CD4^+^ T cells (Jonjic et al., 1989, 1990; Walton et al., 2011).

**Figure 4:**
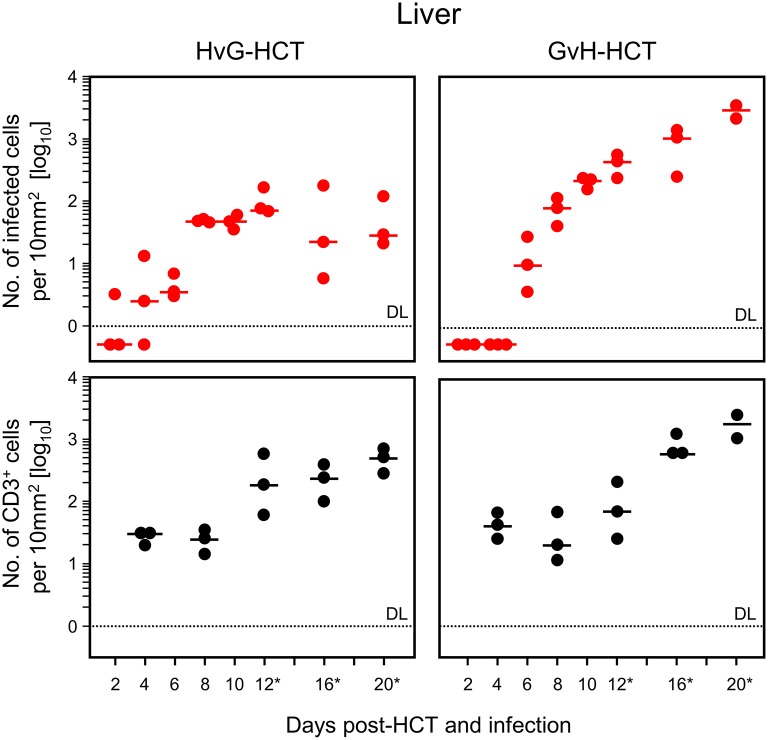
Differential kinetics of virus spread and liver tissue infiltration by T cells after HvG-HCT compared to GvH-HCT. Infected liver cells, which are mostly hepatocytes but also endothelial cells and liver macrophages (Kupffer cells), were identified and quantitated in liver tissue sections by IHC specific for the intranuclear viral IE1 protein (upper panels). Liver-infiltrating T cells were identified and quantitated by IHC specific for the signaling molecule CD3ε of the TCR-CD3 complex (lower panels). Data refer to representative 10-mm^2^ tissue section areas. Symbols represent data for mice tested individually. Median values are marked. Observation times for which histological images are shown in Figure 5 are marked by an asterisk. DL, detection limit of the assay.

**Figure 5:**
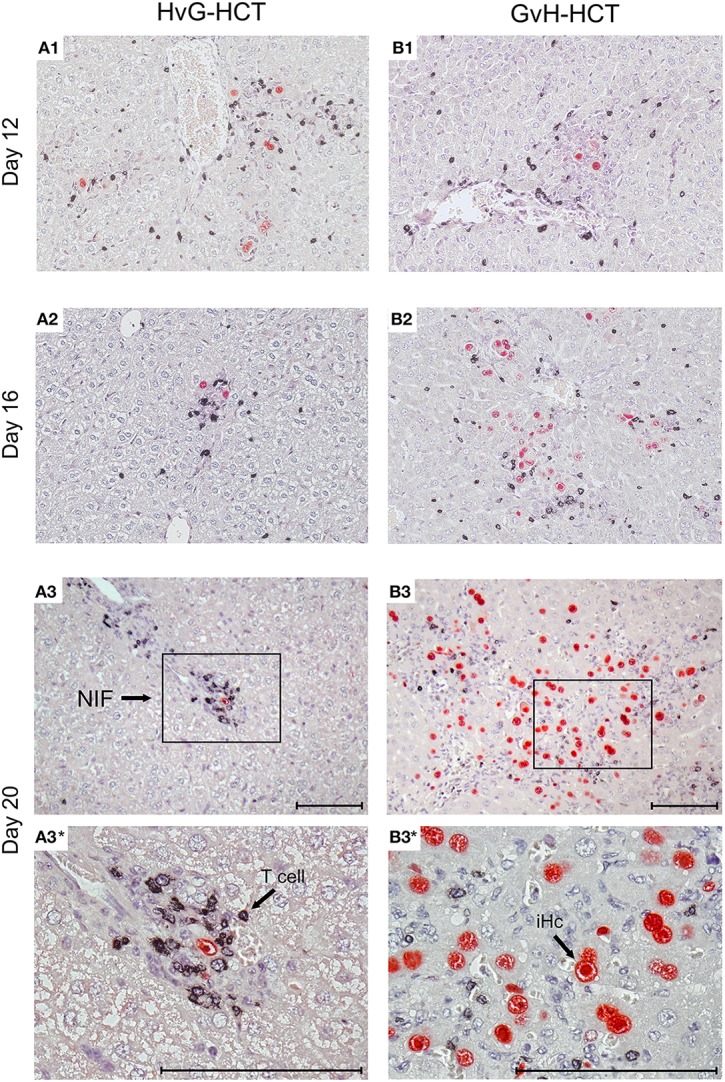
Immunohistological images of liver tissue infection and infiltration by NIF-forming T cells after HvG-HCT compared to GvH-HCT. Representative 2C-IHC images of liver tissue sections are shown corresponding to the quantitative data shown in Figure 4 for the asterisk-marked observation times. (Red IHC staining) IE1 protein in nuclei of infected liver cells. (Black IHC staining) CD3ε protein expressed by T cells. (Left column) HvG-HCT. (Right column) GvH-HCT. (A1–A3 and B1–B3) low magnification overview images. Frames in day-20 images A3 and B3 demarcate regions resolved to greater detail by higher magnification in images A3* and B3*, respectively. NIF, nodular inflammatory focus. T cells in images A3 and A3* cluster around an infected cell to form a NIF that confines the infection after HvG-HCT. Note the disseminated infection and scattered T-cell infiltrates in images B3 and B3* for GvH-HCT. iHc, infected hepatocyte with a typical intranuclear inclusion body that indicates the late phase of the productive viral cycle. Bar markers: 100 μm.

For evaluating viral pathogenesis it is important to relate the burden of infectious virus to viral histopathology (Figure 4, for *bona fide* representative tissue section images, see Figure 5). Although mCMV replicates in multiple organs/tissues of immunocompromised mice after HCT (Podlech et al., 1998), viral histopathology was herein documented with focus on liver infection, because virus spread in liver tissue is best characterized and shows little intra-organ heterogeneity (Podlech et al., 1998; Sacher et al., 2008; Lemmermann et al., 2015). Quantitation of infected liver cells, composed mainly of infected hepatocytes but also of liver sinusoidal endothelial cells and liver macrophages/Kupffer cells (Sacher et al., 2008, 2011; Lemmermann et al., 2015), revealed an increasing difference between HvG-HCT and GvH-HCT over time. Specifically, the numbers of infected hepatocytes stagnate from day 8 onward after HvG-HCT, which indicates onset of immune control on around day 8, whereas after GvH-HCT the infection spreads unhindered. In notable contrast, numbers of liver-infiltrating CD3ε^+^ T cells, which here represent primarily CD8^+^ T cells, were not lower but actually even somewhat higher after GvH-HCT compared to HvG-HCT (Figure 4). This latter finding suggests that, in GvH-HCT, most liver-infiltrating T cells are either not virus-specific or not antivirally functional, and are thus not protective.

The mere quantity of liver-infiltrating CD8^+^ T cells is no reliable indicator for antiviral protection, because only cells that recognize infected cells can contribute to protection. Previous work has revealed a microanatomical correlate of antiviral protection, namely the formation of nodular inflammatory foci (NIF). In these structures, tissue-infiltrating antiviral CD8^+^ T cells congregate at infected cells to prevent intra-tissue virus spread, thereby confining the infection to few tissue cells with no apparent histopathology (Alterio de Goss et al., 1998; Holtappels et al., 1998; Podlech et al., 2000; Sacher et al., 2008; Thomas et al., 2015). Importantly, as shown previously in adoptive cell transfer models with viral epitope-specific CD8^+^ T-cell lines, formation of protective NIF depends on recognition of antigenic peptide presented on infected tissue cells. This was concluded from the finding that NIF were not formed and virus replication was not controlled in mice infected with recombinant mCMV in which the cognate antigenic peptide was functionally deleted by a point mutation affecting the C-terminal MHC class-I anchor amino acid residue (Böhm et al., 2008a; Thomas et al., 2015; Lemmermann and Reddehase, 2016). Thus, absence of NIF formation indicates absence of CD8^+^ T cells capable of recognizing infected cells. In accordance with the established role of NIF in confining the infection, NIF were detected in liver tissue sections from day 16 onward in the case of protective HvG-HCT, whereas in non-protective GvH-HCT, T cells were rarely arranged in NIF but remained randomly distributed in tissue (Figure 5). Note that histological analyses and clinical grading did not reveal classical signs of GvHD in skin, liver, and intestine.

We propose from this set of data that disseminated, cytopathogenic tissue infection and associated organ disease resulting from a failure in the formation of protective NIF is the cause of death in recipients of GvH-HCT.

### GvH-HCT Fails to Generate Sufficiently High Numbers of High-Avidity Antiviral CD8^+^ T Cells

**Figure 6:**
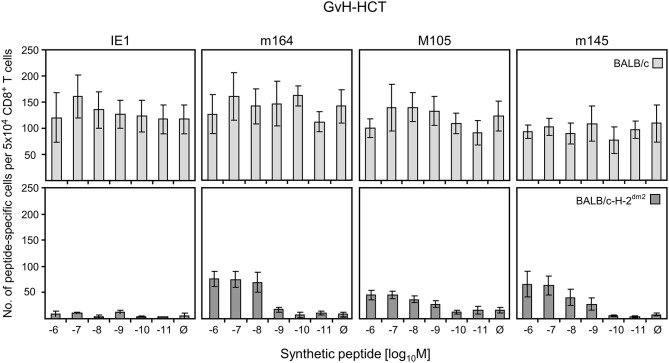
Quantitation of GvH-reactive and viral epitope-specific liver-infiltrating CD8^+^ T cells after GvH-HCT. Liver-infiltrating CD8^+^ T cells were isolated from liver tissue (yield from 10 livers) of infected GvH-HCT recipients on day 20. Their functional activity was tested in an ELISpot assay based on IFNγ secretion in response to stimulation with BALB/c (upper panels) or BALB/c-H-2^dm2^ (lower panels) MEF as target cells that were exogenously loaded with graded loading-concentrations of the indicated synthetic viral peptides to reveal cumulative avidity distributions. Effector cells responding at a certain peptide concentration include cells that respond to concentrations < the indicated concentration. Ø, no viral peptide loaded. Error bars represent 95% confidence intervals determined by intercept-free linear regression.

While the failure in formation of protective NIF explains lethal CMV disease in GvH-HCT, the question remains why the tissue-infiltrating T cells do not form NIF. As NIF formation requires recognition of infected cells that present antigenic viral peptides (referenced above), inefficient reconstitution of viral epitope-specific CD8^+^ T cells after GvH-HCT as well as a failure in antigen presentation could explain missing NIF formation and thus the absence of antiviral function. In addition, previous work has shown that the functional avidity with which CD8^+^ T cells recognize peptide-MHC class-I complexes is decisive for the recognition of infected cells and for protective antiviral activity (Ebert et al., 2012b; Nauerth et al., 2013). We have estimated that CD8^+^ T cells must be able to recognize target cells that are exogenously loaded with synthetic antigenic peptide at a concentration of at least 10^−9^ M in order to detect also infected cells in which MHC class-I molecules are endogenously loaded with naturally processed peptide. We therefore isolated CD8^+^ T cells from infected livers of mice after GvH-HCT to test not just their epitope specificity but also their functional avidities for a panel of immunodominant antigenic peptides of mCMV (Figure 6).

As the target cells in tissues of GvH-HCT recipients are of BALB/c genotype expressing MHC class-I molecules K^d^, D^d^, and L^d^, we first used BALB/c MEF as target cells in an ELISpot assay for quantitating viral epitope-specific CD8^+^ T cells (IE1-L^d^, m164-D^d^, M105-K^d^, and m145-K^d^; Ebert et al., 2012b) in liver infiltrates. Detection is based on IFNγ secretion after sensitization with MEF exogenously loaded with synthetic viral peptides at graded concentrations (Figure 6, upper panels). With this approach, however, it was not possible to identify viral epitope-specific cells, because recognition of unloaded MEF masked a possible viral epitope-specificity. It is not quite unexpected in a GvH-HCT setting that GvH-reactive cells are generated that recognize the mismatch, that is L^d^. L^d^-alloreactive cells mostly recognize L^d^ molecules loaded endogenously with naturally processed cellular peptides. This was concluded from comparing the recognition of L^d^-expressing L^Ld^ cells with that of TAP-deficient T2^Ld^ cells expressing empty MHC class-I complexes (Figure S1).

Next, to disclose viral epitope-specific cells, the liver-infiltrate CD8^+^ T cells were tested with BALB/c-H-2^dm2^ MEF as target cells in the assay, thereby avoiding the response to L^d^ (Figure 6, lower panels). With this approach, CD8^+^ T cells specific for m164-D^d^, M105-K^d^, and m145-K^d^ became visible. However, after background subtraction, almost no cells showed a functional avidity enabling them to recognize peptide at loading concentrations of < 10^−9^ M required for recognition of infected cells and for NIF formation. This low functional avidity of the viral epitope-specific CD8^+^ T-cell population can explain missing control of infection after GvH-HCT.

**Figure 7:**
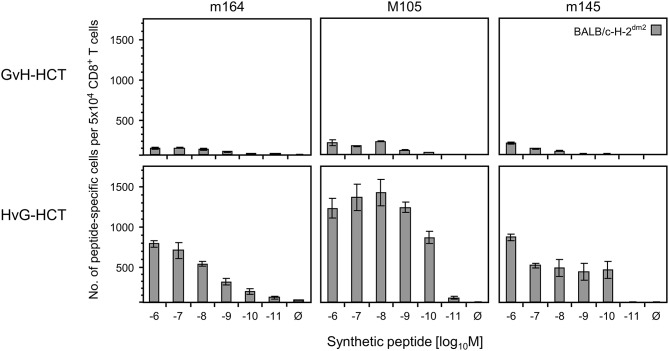
Frequencies of viral epitope-specific liver-infiltrating CD8^+^ T cells after GvH-HCT compared to HvG-HCT. Liver-infiltrating CD8^+^ T cells derived on day 20 from livers (pools of 10 livers per group) of GvH-HCT recipients (upper panels) and HvG-HCT recipients (lower panels) were tested for the recognition of the indicated viral epitopes by using BALB/c-H-2^dm2^ MEF as target cells in order to avoid masking by responses to the L^d^ mismatch. For further details, see the legend to Figure 6.

**Figure 8:**
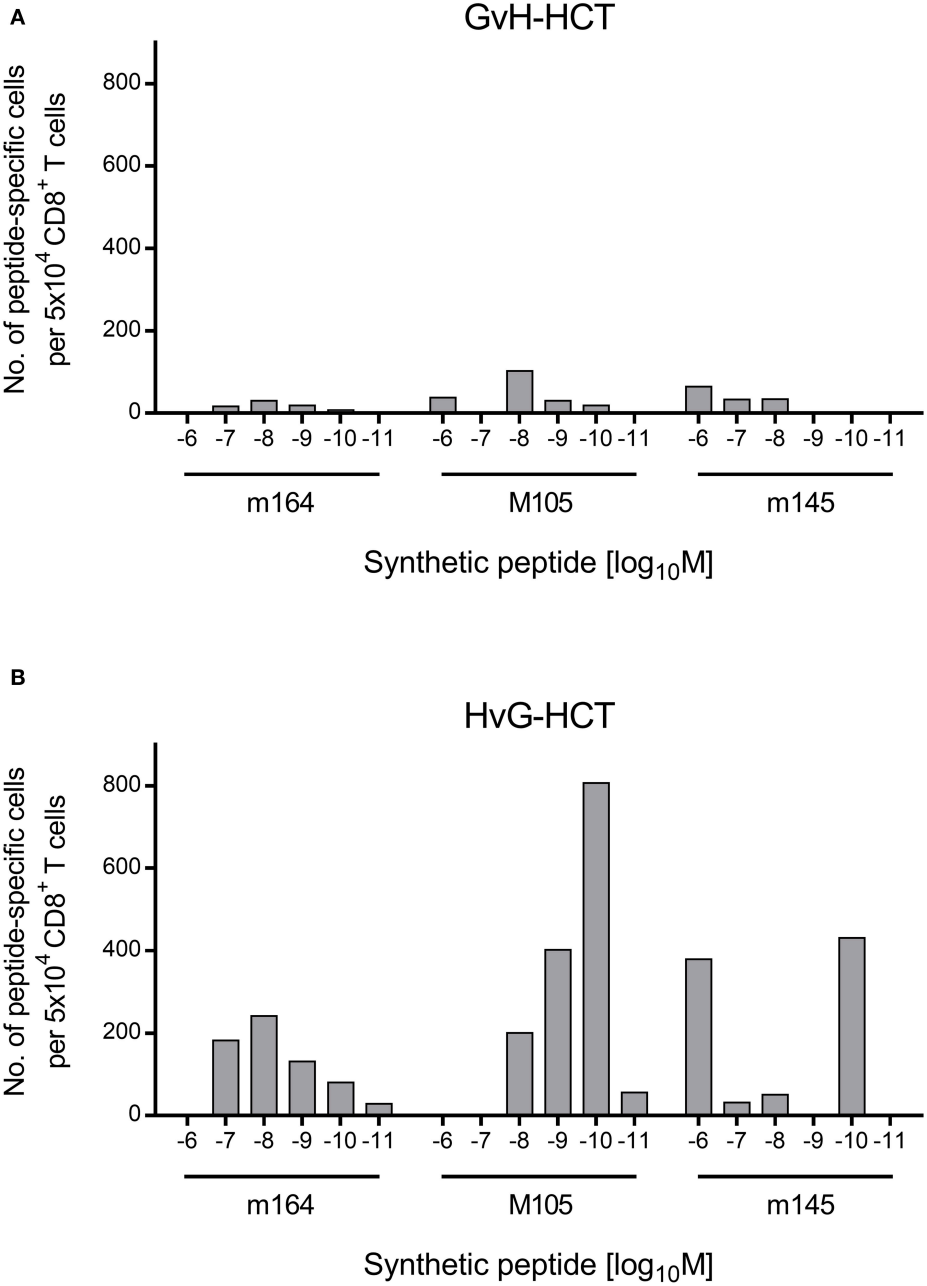
Gaussian-like avidity distributions of liver-infiltrating viral epitope-specific CD8^+^ T cells. **(A)** GvH-HCT. **(B)** HvG-HCT. Data of the experiment shown in Figure 7 are rearranged to reveal the non-cumulative avidity distributions that represent numbers of cells responding precisely to the indicated peptide-loading concentration (for the calculation method, see Holtappels et al., 2008).

If this explanation is valid, NIF-forming CD8^+^ T cells in the liver of HvG-HCT recipients should comprise also high-avidity viral epitope specific cells capable of recognizing presented peptides at loading concentrations of < 10^−9^ M. A direct comparison of liver-infiltrating CD8^+^ T cells after GvH-HCT and HvG-HCT revealed overall higher numbers of viral epitope-specific cells, including high-avidity cells, after HvG-HCT. Specifically, the detection limit in HvG-HCT was 10^−10^ M for the m145 peptide and 10^−11^ M for peptides m164 and M105 (Figure 7). So far, avidities were presented as “cumulative avidity distributions” representing the numbers of cells actually determined to respond when the assay was performed with the indicated molar concentration of peptide. These numbers, logically, include cells that respond also to lower peptide concentrations. The avidity differences are more clearly revealed by the Gaussian-like avidity distributions, which show the numbers of cells that require at least the indicated peptide concentration for responding. This reflects their functional avidity (Figure 8). Obviously, cells with an antiviral functional avidity corresponding to <10^−9^ M peptide are almost missing after GvH-HCT but are frequent after HvG-HCT. It should be emphasized that the data did not provide reliable evidence for an avidity shift to lower avidity caused by the mismatch in GvH-HCT. Rather, the overall decline in numbers of antiviral CD8^+^ T cells leads to vanishingly low numbers of cells with protective functional avidity.

The question might be raised if L^d^-directed alloreactivity in GvH-HCT prevents reconstitution or tissue recruitment of viral epitope-specific CD8^+^ T cells with clonotypic preferences. As shown in experiments that were aimed at characterizing the memory CD8^+^ T-cell pool in the spleen of mCMV-infected BALB/c mice, TCR Vβ chain usage for epitopes IE1 and m164 is quite broad, with distinct but overall rather minor differences to the TCR Vβ chain usage of the total CD8^+^ T-cell population and also among each other. In addition, there is some variance in TCR Vβ chain usage between independent experiments (Figure S2). Specifically, whereas Vβ8.1/8.2 usage dominated throughout, which is in line with a previous report on Vβ chain usage by IE1-specific CD8^+^ T cells (Rodewald et al., 1989), a differential Vβ14 usage is more consistently observed with a preference for the m164 epitope. Notably, previous work on Vβ chain usage revealed the predominance of Vβ8.1/8.2 also for CD8^+^ T cells isolated from the spleens of uninfected BALB/c mice, suggesting that this is a feature specific for this mouse strain rather than a result of mCMV antigen-driven clonal selection (Pahl-Seibert et al., 2005). In fact, in that work, patterns of TCR Vβ usage were quite similar for IE1 epitope-specific memory CD8^+^ cells and overall CD8^+^ T cells in the acute immune response and early or late in the memory phase (Pahl-Seibert et al., 2005). Although we have here not specifically studied Vβ chain usage after GvH-HCT compared to HvG-HCT, the previously observed broad Vβ chain usage in the BALB/c model in combination with an almost complete absence of tissue-infiltrating CD8^+^ T cells specific for three epitopes argues against a preferential loss of particular clonotypes. It rather suggests an epitope- and clonotype-independent, more global interference of MHC class-I mismatch with the antiviral immune response after GvH-HCT.

### Immune Evasion Proteins Dictate the Functional Avidity Required for Antiviral Protection

So far, data strongly suggest that lethal infection after GvH-HCT results from the inability of low-avidity antiviral CD8^+^ T cells to recognize infected cells and to form NIF that prevent disseminated cytopathogenic infection. Yet, at this stage of investigation, a critical contribution of the randomly-distributed alloreactive CD8^+^ T cells directed against cellular peptides, presented by the mismatch molecule L^d^ on uninfected tissue cells, was not formally excluded.

Enhancing antigen presentation in infected tissue cells for recruiting low-avidity CD8^+^ T cells into NIF should provide clarification. High-avidity is required for recognition of infected cells because viral immune evasion proteins, m06/gp48 (Reusch et al., 1999; Fink et al., 2019) and m152 glycosylation isoforms p36 and gp40 (Ziegler et al., 1997; Krmpotic et al., 1999; Holtappels et al., 2004; Fink et al., 2013) in the specific case of mCMV, strongly limit the presentation of antigenic peptides (for reviews, see Reddehase, 2002; Lemmermann et al., 2012). Few “escapees” that nevertheless reach the cell surface can only be detected by high-avidity CD8^+^ T cells (Ebert et al., 2012b). Alleviation of immune evasion by infection with a recombinant virus in which immune evasion genes, also referred to as “viral regulators of antigen presentation” (vRAP) (Holtappels et al., 2006), are deleted (Wagner et al., 2002) should recruit also low-avidity CD8^+^ T cells into protective NIF.

**Figure 9:**
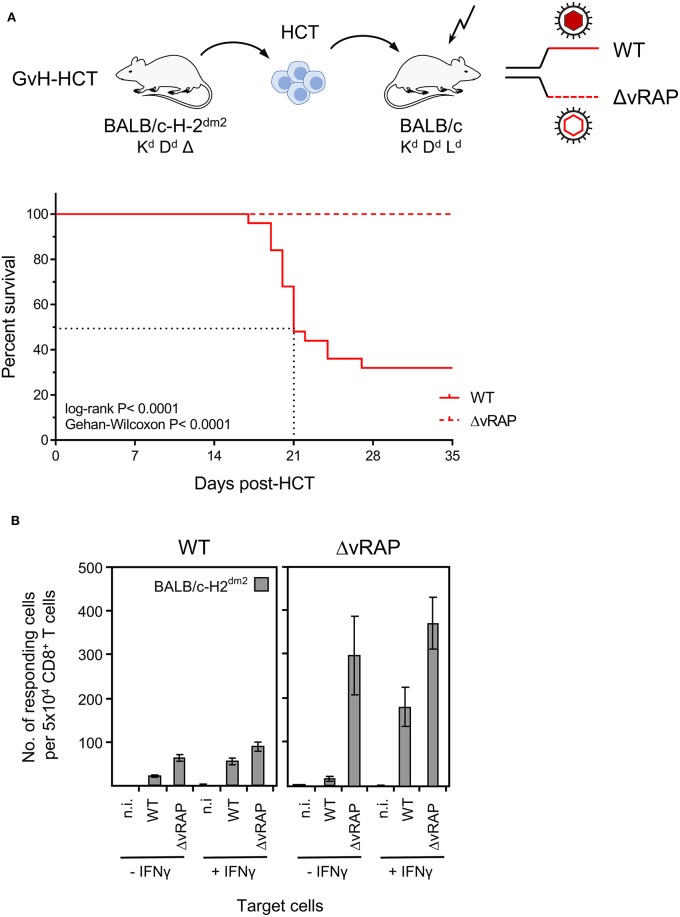
Rescue of GvH-HCT recipients by deletion of immune evasion genes for improving antigen presentation. **(A)** Sketch of the GvH-HCT protocol (top) and resulting Kaplan-Meyer survival plots after infection of the recipients with wild-type (WT) virus or a virus in which ‘viral regulators of antigen presentation’ (vRAP) are deleted (ΔvRAP). **(B)** Improvement of antigen presentation by deletion of vRAP or by IFNγ. Liver-infiltrating CD8^+^ T cells derived on day 20 from livers (pools of 10 livers per group) of GvH-HCT recipients infected with WT virus (left panel) or ΔvRAP virus (right panel) were tested in the IFNγ-based ELISpot assay for recognition of BALB/c-H-2^dm2^ MEF that were either left uninfected (n.i., not infected) or were infected with either WT virus or ΔvRAP virus. One set of these three types of target cells was pre-treated with IFNγ (+IFNγ), another set was left untreated (-IFNγ). Bars represent numbers of responding cells. Error bars represent 95% confidence intervals determined by intercept-free linear regression.

The result of the corresponding experiment gives a clear answer (Figure 9A). After GvH-HCT and infection with wild-type (WT) virus expressing vRAP, high lethality was reproduced, and again the median survival time was 21 days (recall Figure 1). In contrast, under otherwise identical transplantation conditions, all mice survived in the same experiment when infected with ΔvRAP virus. In accordance with the known vRAP function, somewhat more CD8^+^ T cells isolated from WT virus-infected GvH-HCT recipients recognized target cells infected *in vitro* with ΔvRAP virus compared to cells infected with WT virus. This difference was far more pronounced for liver-infiltrating CD8^+^ T cells isolated from GvH-HCT recipients that were infected with the ΔvRAP virus (Figure 9B).

As an alternative to vRAP deletion in the infecting virus, antigen presentation can also be enhanced in infected cells by IFNγ that counteracts vRAP function. Importantly, in previous work, adoptive transfer of virus-specific CD8^+^ T cells protected against vRAP-expressing WT mCMV in immunocompromised IFNγ-transgenic B6-SAP-IFNγ mice that constitutively produce IFNγ in the liver, whereas the same cells failed to protect non-transgenic littermates in which systemic IFNγ levels are low (Fink et al., 2012). It should be emphasized that IFNγ produced in immunocompromised B6-SAP-IFNγ mice did not reduce replication of WT mCMV directly, which indicates that the antiviral function of IFNγ is exerted by enhancing antigen presentation to antiviral CD8^+^ T effector cells (Fink et al., 2012). The poor recognition of WT mCMV-infected tissue cells after GvH-HCT thus also indicates that IFNγ was not sufficiently available locally at the infected cells to override vRAP function *in vivo*. The potential of IFNγ to enhance antigen presentation despite the presence of vRAP is documented by *in vitro* recognition of IFNγ-pretreated cells infected with vRAP-expressing WT virus. Again, the number of responding cells was higher when the GvH-HCT recipients had been infected with ΔvRAP virus. IFNγ could not further improve the recognition of cells in which antigen presentation was already optimal after *in vitro* infection with ΔvRAP virus (Figure 9B).

**Figure 10:**
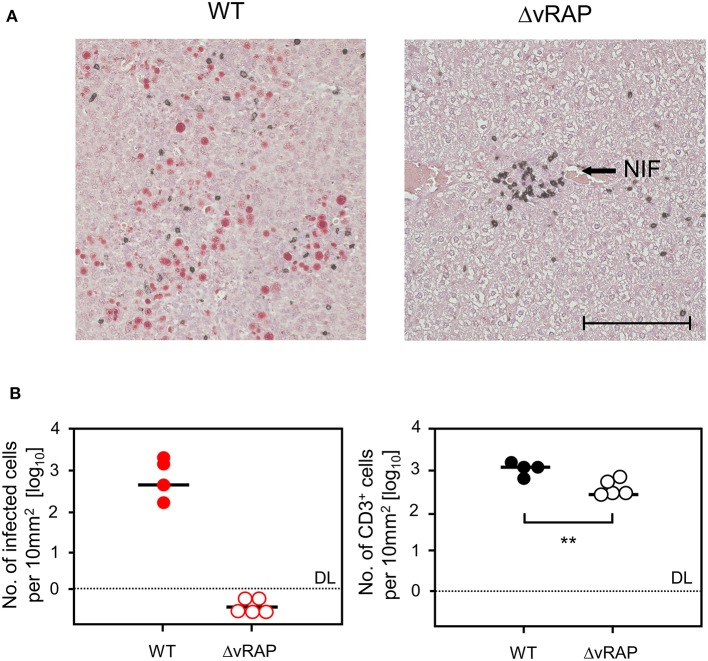
Immunohistological analysis of liver tissue infection and infiltration by T cells in GvH-HCT recipients depending on immune evasion. Liver tissue sections were taken on day 20 after GvH-HCT and infection of the recipients with either WT virus (left panels) or ΔvRAP virus (right panels), corresponding to the experiment shown in Figure 9. **(A)** Representative 2C-IHC images with red staining of IE1 protein in nuclei of infected liver cells and black staining of CD3ε protein expressed by T cells. NIF, nodular inflammatory focus. Bar marker: 100 μm. **(B)** Quantitation of infected, IE1^+^ liver cells (left panel) and of liver-infiltrating CD3ε^+^ T cells (right panel). Data refer to representative 10-mm^2^ tissue section areas. Symbols represent recipients tested individually. Median values are marked. DL, detection limit of the assay. Statistical analysis is performed by Student's *t*-test based on log-transformed data. Significance level: *P*-value of <0.01 (**).

These findings show that efficient *in vivo* antigen presentation on infected tissue cells not only improves recognition for the antiviral effector phase but also recruits more virus-specific CD8^+^ T cells to the sites of infection, likely stimulating also their on-site proliferation. Again, the histopathological correlate of lethality was an extensive virus spread in the case of infection with WT virus, whereas after infection with ΔvRAP virus protective NIF were formed and prevented virus spread and viral histopathology (Figure 10A). This was the case despite a lower overall tissue infiltration by T cells after infection with ΔvRAP virus, which can be explained by less inflammation as a result of efficient control of the infection (Figure 10B).

Collectively, the data give reasonable evidence to conclude that lethality from mCMV infection under conditions of GvH-HCT is caused by a failure to mount a high-avidity CD8^+^ T-cell response capable of recognizing infected tissue cells in which the presentation of viral antigens is limited by viral immune evasion.

## Discussion

Most previous studies in the mouse model of experimental HCT and concomitant acute infection, modeling a clinical scenario of early reactivation of latent hCMV post-HCT, have addressed the clinical setting of syngeneic HCT by using the mouse strain BALB/c (MHC haplotype *H-2*^*d*^) as HCT donor and recipient (reviewed in Holtappels et al., 2013; Reddehase, 2016). In this model, control of virus replication and prevention of lethal CMV disease in the transiently immunocompromised HCT recipients proved to be based on rapid lympho-hematopoietic reconstitution of antiviral CD8^+^ T cells that infiltrate infected tissues and confine the infection to NIF before an extensive viral histopathology can lead to organ failure (Holtappels et al., 1998; Podlech et al., 1998, 2000). Accordingly, delayed reconstitution by transplantation of too low numbers of HC as well as depletion of CD8^+^ T cells in the phase of reconstitution is associated with disseminated tissue infection. This leads to multiple organ CMV disease manifestations and death even when donor and recipient are identical in all MHC and non-MHC antigens. A quantitatively insufficient HCT transplant has been shown to allow mCMV to infect bone marrow stroma. This leads to reduced expression of essential hemopoietins and thereby inhibits reconstitution of cells of all hematopoietic lineages, resulting in a prolonged “window of risk” due to maintained immunodeficiency (Mayer et al., 1997; Steffens et al., 1998; Renzaho et al., 2020).

In allogeneic clinical HCT with family donors or unrelated donors, partial mismatches in transplantation antigens are unavoidable and bear a risk of developing GvHD and, depending on residual T cells in the recipient, also a risk of graft rejection by HvG reactivity. Previous work has introduced the model of experimental HCT with a singular MHC class-I mismatch (Alterio de Goss et al., 1998; Seckert et al., 2011), the model also used here. This model has the specific advantage that HvG and GvH complications in allogeneic HCT can be studied separately (recall Figure 1A). In the HvG setting with BALB/c donors expressing the MHC class-I molecule L^d^ and with BALB/c-H-2^dm2^ recipients not-expressing L^d^, most recipients survived mCMV infection, although lethality was somewhat higher than in syngeneic HCT with BALB/c donors and recipients (Alterio de Goss et al., 1998). As these two types of HCT share the donor mouse strain, the hematopoietic potential of the transplanted donor HC cannot provide an explanation for the observed 1-week delay in tissue infiltration and control of infection after HvG-HCT compared to syngeneic HCT (Alterio de Goss et al., 1998; Holtappels et al., 1998). Here we have studied the opposite direction of transplantation, namely GvH-HCT with BALB/c-H-2^dm2^ mice as donors that do not express L^d^ and with BALB/c mice as recipients that express L^d^, so that the immunogenetic condition for a potential GvH reactivity is fulfilled.

A pathogenetic link between GvHD and mCMV infection has previously been studied in a parent-into-F1 mouse model with a bidirectional “MHC class-I only” donor-recipient disparity, arriving at the conclusion that mCMV infection enhances GvHD (Cray and Levy, 1989, 1993; Via et al., 1990). This finding, though interesting and to some extent related to our work, was not predictive for the GvH-HCT model studied here, because GvHD was induced by transfer of high doses of mature parent donor T cells into immunocompetent F1 recipients. This is a setting essentially different to HCT, where donor cells are HC cells that need to reconstitute recipients immunocompromised by hematoablative treatment. More related to our work is a parent-into-F1 model of allogeneic HCT in which chronic GvHD at > 100 days after HCT was found to be associated with an impaired antigen-specific antiviral immune response that was characterized by impaired tissue homing of antigen-specific T cells (Hossain et al., 2007). Besides a discussed mutual exacerbation of disease severity, evidence has been provided for a role of GvH and HvG responses in increasing the incidence of CMV reactivation from latency (Söderberg-Nauclér et al., 1997; Zhang et al., 2019).

We have here found an impaired control of tissue infection that is associated with lethality in an acute disease model of GvH-HCT and simultaneous infection with mCMV in absence of apparent GvHD-characteristic histopathology. Tissue infiltrates consisting of CD8^+^ T cells were characterized by disseminated cells specific for the mismatch, that is for L^d^ or cellular peptides presented by L^d^ in the specific example, as well as of reduced overall numbers and almost absence of high-avidity virus-specific CD8^+^ T cells. In contrast to HvG-HCT, where high numbers of high-avidity antiviral CD8^+^ T cells recognized infected tissue cells and formed protective NIF that confined and eventually terminated the infection, the few and mainly low-avidity tissue-infiltrating antiviral CD8^+^ T cells after GvH-HCT failed to form protective NIF. This resulted in disseminated tissue infection and thus in viral histopathology. The finding that enhancement of antigen presentation in infected cells by deletion of viral immune evasion genes reverts the outcome of GvH-HCT to survival provides evidence for the conclusion that death after infection with WT virus is caused by viral histopathology and not by GvH reaction directed against uninfected tissue cells of the recipients.

The question remains why HC derived from L^d^-deficient BALB/c-H-2^dm2^ donors reconstituted antiviral CD8^+^ T cells so inefficiently. One immediate suspicion might be that BALB/c-H-2^dm2^ mice are “low responders” in general or specifically to mCMV. This possibility can be refuted based on previous work having shown that the acute and memory CD8^+^ T-cell response to mCMV is absolutely comparable in the two mouse strains, with the trivial exception that a response to L^d^-restricted viral antigenic peptides is absent in the *L*^*d*^ gene deletion mutant BALB/c-H-2^dm2^ (Seckert et al., 2011). A gap in the TCR repertoire of BALB/c-H-2^dm2^ donors preventing the recognition of antigenic peptides presented by L^d^ on cells of BALB/c recipients cannot explain the general response deficiency that includes responses to antigenic peptides presented by K^d^ and D^d^ shared by the two mouse strains. The idea that a contribution by L^d^-restricted CD8^+^ T cells is quantitatively or qualitatively critical for antiviral protection is also untenable in face of the finding that the infection is controlled in HvG-HCT in which the recipients cannot present L^d^-restricted antigenic peptides (Alterio de Goss et al., 1998; this report). Finally, protective activity of polyclonal mCMV-specific CD8^+^ T cells is so redundant that even deletion of immunodominant antigenic peptides presented by K^d^, D^d^, and L^d^ is tolerated (Holtappels et al., 2008, 2016; Ebert et al., 2012a).

Killing of radiation-resistant antigen-presenting cells of recipient genotype by GvH-reactive L^d^-specific CD8^+^ T cells can be discussed as a putative mechanism compatible with the finding that immunodeficiency in GvH-HCT affects also responses against viral peptides presented by the shared MHC class-I molecules K^d^ and D^d^. However, previous work has shown that after sex-mismatched syngeneic HCT, resulting in mixed chimeras, recipient-resident (*sry*^−^) CD11c^+^ dendritic cells (DC) are completely replaced with donor-derived (sry^+^) DC, whereas cells of other hematopoietic lineages, for instance CD11b^+^ cells, are chimeric (Seckert et al., 2009). Likewise, donor origin of CD11c^+^ DC was also shown after HvG-HCT by cytofluorometric detection of L^d^ (Seckert et al., 2011). So, antigen-presenting DC in GvH-HCT are of BALB/c-H-2^dm2^ genotype not expressing L^d^. With the same argument, competition between viral epitope-specific naïve CD8^+^ T cells and mismatch-specific naïve CD8^+^ T cells for priming by DC is refuted either. Future experiments will have to address the question if a GvH response or a histoincompatibility as such generates a tolerizing cytokine micromilieu or induces regulatory T cells that inhibit priming, proliferation, or tissue-recruitment of antiviral CD8^+^ T cells.

The here studied model is of great scientific value, because it allowed separating GvH and HvG complications of allogeneic HCT. This is based on the experimental strategy making use of a mismatch caused by genetic deletion of an MHC class-I molecule in the mouse mutant BALB/c-H-2^dm2^ chosen as HCT donor or recipient, respectively. This advantage, however, might also be seen as a limitation, because mismatch by gene deletion has no obvious correlate in clinical HCT. As we show in an alternative model of GvH-HCT with a donor (C57BL/6; *H-2*^*b*^) vs. recipient (BALB.B; *H-2*^*b*^) match in MHC antigens but mismatch in minor histocompatibility antigens (minor-HAg), GvH-associated lethality of mCMV infection and its prevention by enhanced viral antigen presentation can be reproduced (Gezinir et al., 2020).

**Figure 11:**
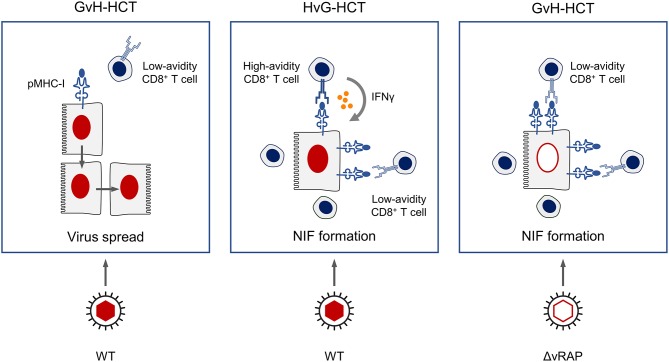
Graphical summary.

Regarding the scenario in the antiviral effector phase in infected host tissues, we propose a key role of the functional avidity of virus-specific CD8^+^ T cells in infected-cell recognition, NIF formation, and control of intra-tissue virus spread (Figure 11). In GvH-HCT and infection with WT virus, low numbers of low-avidity CD8^+^ T cells fail to recognize target cells in which antigen presentation is largely inhibited by viral immune evasion mechanisms. Accordingly, they do not become stimulated, NIF are not formed, and infection spreads uncontrolled. In HvG-HCT and infection with WT virus, sufficient numbers of high-avidity CD8^+^ T cells are able to recognize even limited antigen presentation and thereby become stimulated to secrete IFNγ that relieves immune evasion. The thereby enhanced antigen presentation then recruits also low-avidity CD8^+^ T cells into protective NIF. In GvH-HCT recipients infected with the immune evasion gene deletion mutant ΔvRAP, uninhibited antigen presentation can be recognized also by low-avidity CD8^+^ T cells that form NIF and control the infection.

## Data Availability Statement

The datasets generated for this study are available on request to the corresponding author.

## Ethics Statement

The animal study was reviewed and approved by the ethics committee of the Landesuntersuchungsamt Rheinland-Pfalz according to German federal law §8 Abs. 1 TierSchG (animal protection law), permission numbers 177-07/941-4, 177-07/931-17, 177-07-04/051-62 and 177-07/G09-1-004.

## Author Contributions

RH and MR designed the study. RH, MR, and NL are responsible for the analysis and interpretation of the data. RH, SS, OO, JP, and CS conducted the work and analyzed the data. MR wrote the first draft of the manuscript. RH and NL wrote sections of the manuscript. All authors contributed to manuscript revision, read and approved the submitted version.

### Conflict of Interest

The authors declare that the research was conducted in the absence of any commercial or financial relationships that could be construed as a potential conflict of interest.

## Supplementary Material

Figure S1 GvH-reactive CD8^+^ T cells recognize MHC class-I molecules that present cellular peptides. A cytolytic T-lymphocyte (CTL) line was raised by stimulating BALB/c-H2^dm2^ (L^d^-negative) spleen cells with BALB/c (L^d^-positive) spleen cells in a classical “mixed lymphocyte reaction.” After two rounds of stimulation, cells of the short-term line were tested as effector cells for the recognition of TAP-sufficient (L and L^Ld^) or TAP-deficient (T2 and T2^Ld^) target cells. **(A)** Percentage of cells of the CTL line that respond with IFNγ secretion in an ELISpot assay. Error bars represent 95% confidence intervals determined by intercept-free linear regression. **(B)** Cytolytic activity determined in a [Cr^51^]-release assay at the indicated effector-to-target cell (E:T) ratios.

Figure S2 Patterns of TCR Vβ chain usage in CD8^+^ memory T-cell populations. **(A,B)**, identically performed experiments revealing Vβ chain usage patterns in immunomagnetically-purified, spleen-derived CD8^+^ T cells at 7 or 8 months, respectively, after intraplantar mCMV infection. (Top panels, all TCR^+^) Cytofluorometric analysis of Vβx expression by all CD8^+^ T cells. (Center panels, IE1-TCR^+^) Gating on cells stained with IE1 peptide-L^d^ multimers. (Bottom panels, m164-TCR^+^) Gating on cells stained with m164 peptide-D^d^ multimers. Bars and numbers show the percentages of cells expressing the indicated Vβ chains.
