# Supplementary figures and images for: Insufficient Antigen Presentation Due to Viral Immune Evasion Explains Lethal Cytomegalovirus Organ Disease After Allogeneic Hematopoietic Cell Transplantation
Source: Front Cell Infect Microbiol. 2020 Apr 15;10:157. doi: 10.3389/fcimb.2020.00157 (PMC7174590; doi:10.3389/fcimb.2020.00157)

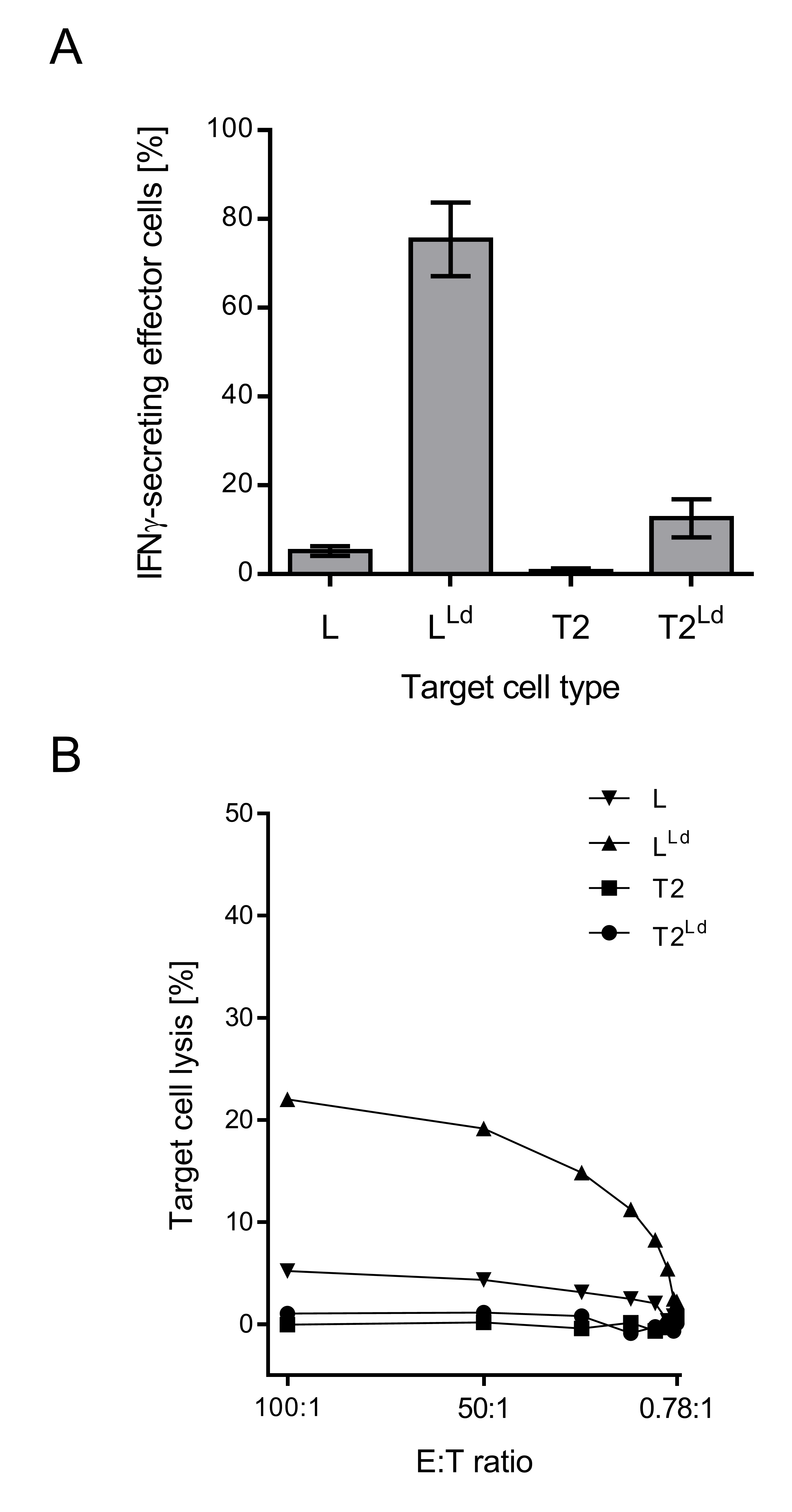

Supplement: Figure S1 — GvH-reactive CD8+ T cells recognize MHC class-I molecules that present cellular peptides. A cytolytic T-lymphocyte (CTL) line was raised by stimulating BALB/c-H2dm2 (Ld-negative) spleen cells with BALB/c (Ld-positive) spleen cells in a classical “mixed lymphocyte reaction.” After two rounds of stimulation, cells of the short-term line were tested as effector cells for the recognition of TAP-sufficient (L and LLd) or TAP-deficient (T2 and T2Ld) target cells. (A) Percentage of cells of the CTL line that respond with IFNγ secretion in an ELISpot assay. Error bars represent 95% confidence intervals determined by intercept-free linear regression. (B) Cytolytic activity determined in a [Cr51]-release assay at the indicated effector-to-target cell (E:T) ratios. [file Image_1.TIF]

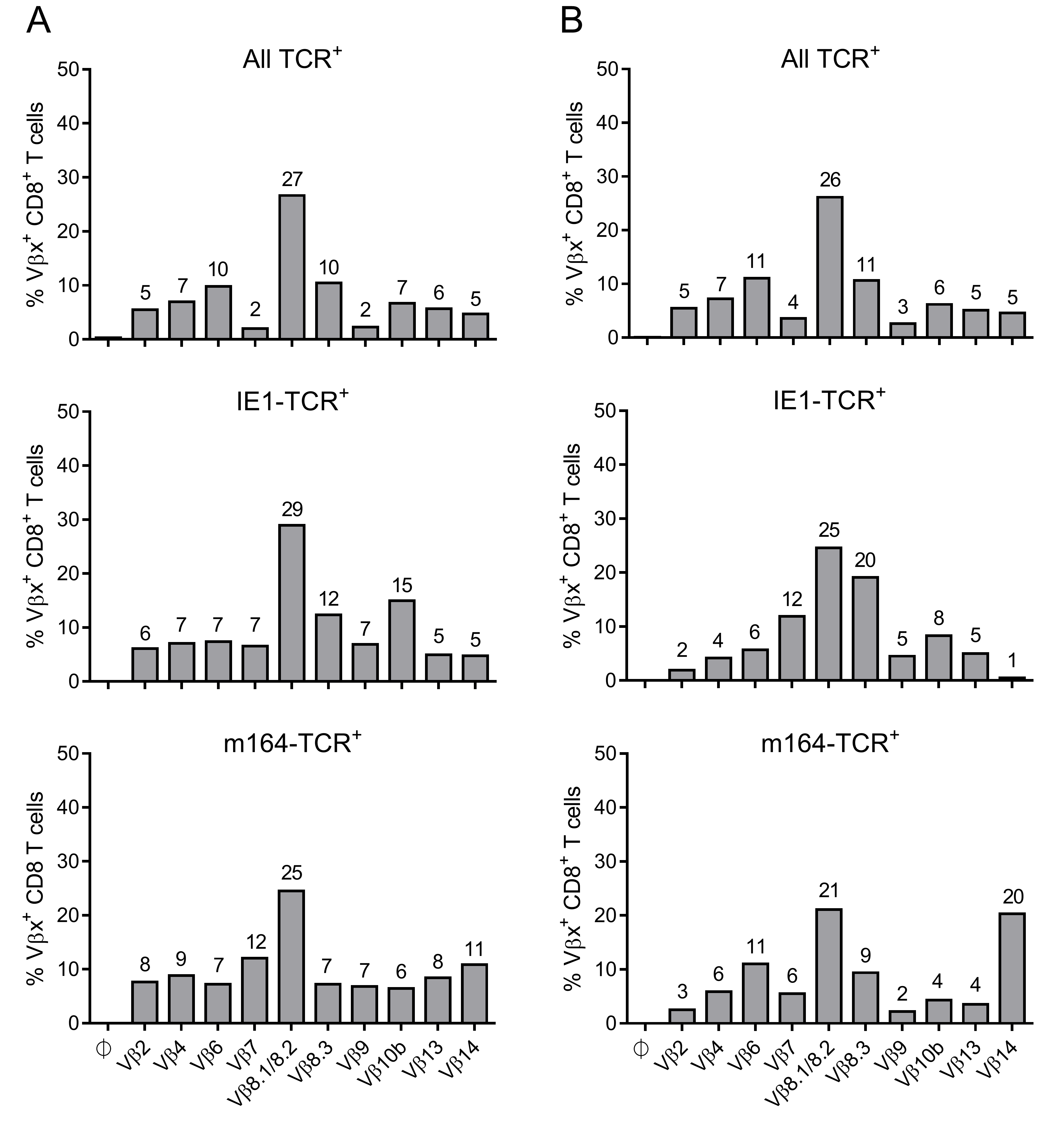

Supplement: Figure S2 — Patterns of TCR Vβ chain usage in CD8+ memory T-cell populations. (A,B), identically performed experiments revealing Vβ chain usage patterns in immunomagnetically-purified, spleen-derived CD8+ T cells at 7 or 8 months, respectively, after intraplantar mCMV infection. (Top panels, all TCR+) Cytofluorometric analysis of Vβx expression by all CD8+ T cells. (Center panels, IE1-TCR+) Gating on cells stained with IE1 peptide-Ld multimers. (Bottom panels, m164-TCR+) Gating on cells stained with m164 peptide-Dd multimers. Bars and numbers show the percentages of cells expressing the indicated Vβ chains. [file Image_2.TIF]
